# *Legionella* in Water Systems: New Frontiers in Detection, Prevention, and Public Health Strategies

**DOI:** 10.3390/microorganisms14091992

**Published:** 2026-09-09

**Authors:** Saleh A. Aloraini

**Affiliations:** Department of Civil Engineering, College of Engineering, Qassim University, Buraydah 52571, Saudi Arabia; saleh.aloraini@qu.edu.sa

**Keywords:** *Legionella pneumophila*, premise plumbing, biofilm, viable but non culturable, digital droplet PCR, secondary disinfection, evidence appraisal, water safety plans

## Abstract

Legionnaires’ disease incidence has risen substantially across industrialised nations since the early 2000s, yet standard control protocols—primarily secondary disinfection verified by culture—rely on an evidence base that warrants further scrutiny. This review offers three interpretations of the available evidence, without claiming they are established conclusions. First, culture-based verification has inherent methodological limitations: treatments that reduce culturability can induce viable-but-non-culturable (VBNC) states undetected by culture alone, creating potential discrepancies between verified culture reduction and complete microbial clearance. This reading rests on evidence that monochloramine at ≥1 mg/L abolishes culturability while genomic units remain essentially unchanged and that starved VBNC *Legionella* retain the capacity to infect human macrophages after more than 220 days, albeit at roughly 1% the efficiency of culturable cells; whether this translates into material risk in operating buildings remains undetermined. Second, much of the comparative-effectiveness literature evaluating disinfectants relies on observational single-site studies, and early validation frameworks often depended on self-reported survey data; randomised comparison in occupied healthcare buildings is frequently impractical, though recent systematic reviews now supply quantitative synthesis earlier work lacked. Third, regulatory thresholds remain expressed in the units of the method whose deficiencies motivated the alternatives, and the alignment of verification standards with advances in molecular detection remains an ongoing challenge.

## 1. Introduction and Epidemiological Drivers

Legionnaires’ disease is a disease of engineered water systems. Its control literature developed largely through the work of a small number of research groups in a small number of hospitals, under conditions in which randomised comparison was neither practical nor, in acute outbreak settings, ethical [1,2]. The influence of that origin on current practice is a principal concern of this review.

### 1.1. The Paradox of Rising Incidence

Reported incidence has increased substantially across North America and Europe since approximately 2000 [3,4]. Dietersdorfer et al. note 7034 notified cases across the EU, Norway, Iceland and Switzerland in 2015, of which 81% of culture-confirmed cases were *Legionella pneumophila* serogroup 1, while cautioning that low notification rates in some countries mean these figures understate true incidence [5].

That caveat can now be stated quantitatively rather than in general terms because the diagnostic literature characterises the relevant ascertainment properties. Bai et al. report that approximately 82% of European Legionnaires’ disease cases are confirmed by urinary antigen test (UAT) and that UAT detects only serotype 1, with sensitivity of 55–80% and specificity of 100% [6]. Culture, the nominal gold standard, has a sensitivity of only 60–80% and requires qualified respiratory specimens that fewer than half of patients produce—about 16% of patients present without cough at all and 38% with dry cough [6]. PCR sensitivity is reported across a strikingly wide range of 17–100% [6].

**Figure 1:**
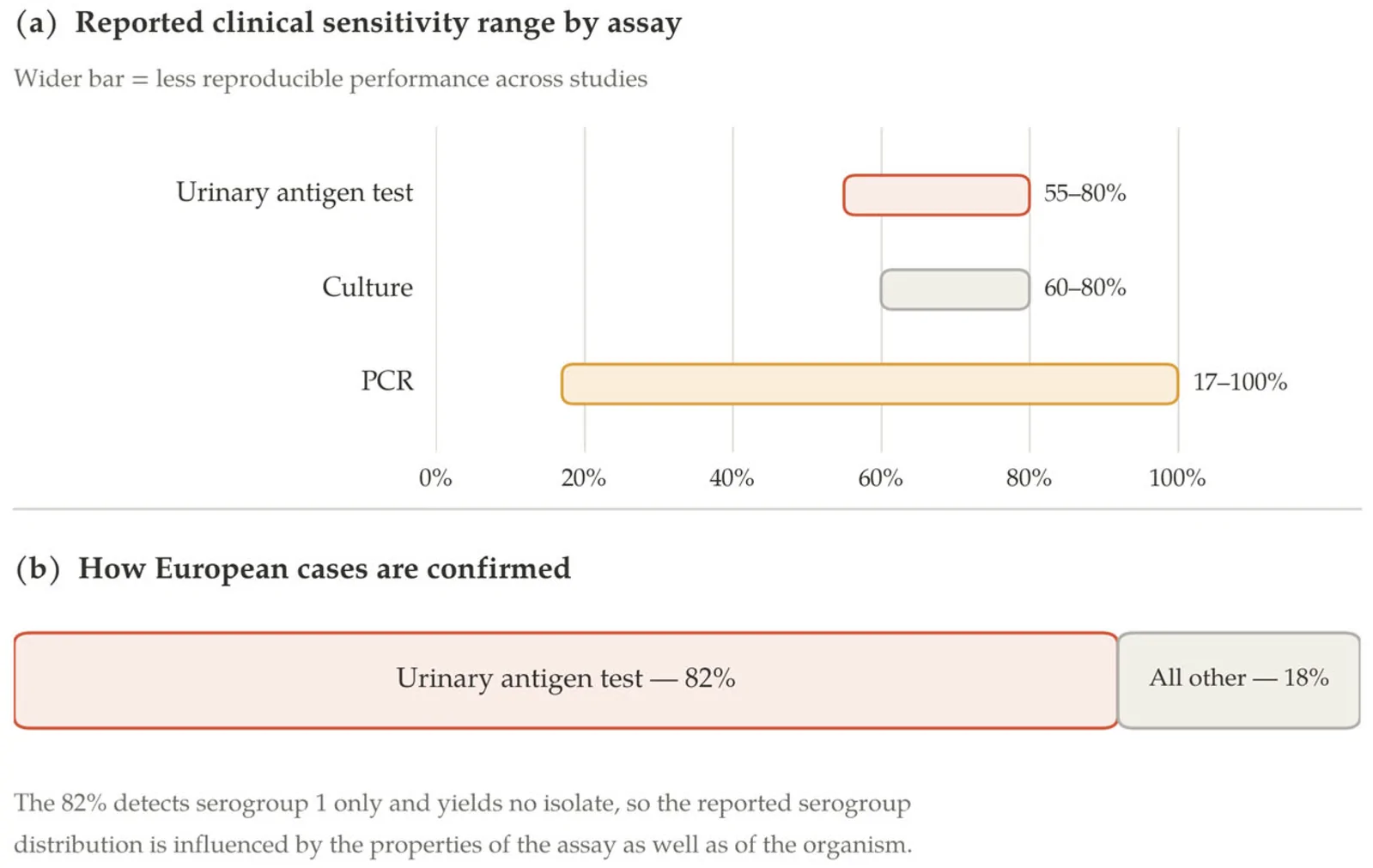
The ascertainment aperture through which *Legionella* epidemiology is observed. Values obtained from Bai et al. [6].

A structural consequence follows. A surveillance system in which four-fifths of confirmed cases are ascertained by a serogroup-1-specific assay will tend to report a serogroup-1-dominated epidemiology, and the reported distribution cannot readily be separated from the properties of the assay. The apparent epidemiology of *Legionella* may therefore be influenced, in part, by the diagnostic methods employed—a theme that recurs throughout this review and is discussed further in the environmental monitoring literature of Section 2 and Section 3. However, diagnostic ascertainment is only one plausible contributor among several: population ageing, expanded use of immunosuppressive therapy, increased clinician awareness and testing intensity, ageing distribution infrastructure and climatic trends have each been proposed, and the available surveillance data do not permit their relative contributions to be partitioned [3,4,7,8,9]. Accordingly, the non-*pneumophila* and non-serogroup-1 disease burden may be currently under-characterized rather than demonstrably minimal. The magnitude is documented directly: age-standardised average incidence in the United States rose from 0.48 cases/100,000 population during 1992–2002 to 2.71 cases/100,000 in 2018, corresponding to an incidence risk ratio of 5.67 (95% CI of 5.52–5.83), with an average annual increase of 9.3% (95% CI of 8.1–10.4%) from 2002 onwards [3]. Figure 1 sets the reported performance of the three modalities against the share of cases each ascertains.

### 1.2. Host Susceptibility and Demographic Transition

Legionnaires’ disease is overwhelmingly a disease of the susceptible host. Population-attributable risk is dominated by age above 50, current or former smoking, chronic obstructive pulmonary disease, diabetes, malignancy, chronic kidney and liver disease, and—most acutely—pharmacological immunosuppression [7,10,11]. Bai et al. list the same risk profile: age, gender, smoking, alcoholism, immunosuppression, and comorbid chronic cardiopulmonary disease [6].

This matters epidemiologically because susceptibility interacts multiplicatively with exposure. A hospital water system delivering a given *Legionella* concentration poses a categorically different risk in a transplant unit than in an administrative building. It also means population ageing alone predicts continued incidence growth, independent of any change in environmental *Legionella* burden—a sobering consideration for programmes that measure success in environmental terms.

### 1.3. Ageing Distribution Infrastructure

The public distribution network is the upstream reservoir that seeds premise plumbing. Several deficits are consequential: disinfectant residual decay in oversized, low-demand mains; main breaks and pressure transients permitting intrusion and mobilising accumulated biofilm; iron corrosion generating particulate oxides that consume residual and supply nutrients supporting *Legionella*-hosting biofilm; and treatment chemistry changes that perturb an established distribution microbiome [7,12,13].

### 1.4. Climatic Drivers

Meteorological associations with Legionnaires’ disease are among the more robust in environmental epidemiology, with elevated risk following increased precipitation and high relative humidity, typically at one- to two-week lags [8,9,14]. Dietersdorfer et al. cite an increased need for conditioned water under climate change as a driver of rising incidence [5], and Bai et al. identify warm, humid soil and summer seasonality among the epidemiological characteristics [6]. Plausible mechanisms include enhanced aerosol persistence under high humidity, elevated ambient temperature raising cold-water plumbing into the permissive range, and increased cooling-tower duty cycles during heat events.

### 1.5. Premise Plumbing: The Critical Control Domain

The final tens to hundreds of metres of the supply chain generate most of the risk for reasons that are largely geometric and thermal: a high wetted-surface-to-volume ratio, delivery temperatures moderated into the *Legionella* growth optimum for scald prevention, extensive stagnation, hydraulic complexity, and materials that leach assimilable organic carbon [7,15,16].

Margot et al. provide unusually direct evidence on the materials point. Working with EPDM rubber—the elastomer used in sealing rings, chosen precisely because it is known to leach assimilable organic carbon and support colonisation—they observed total organic carbon in the water phase rise from 2.0 mg/L at experiment start to 3.4 mg/L by week 3, then fall to 1.4 mg/L by week 7, consistent with a finite leachable carbon pool driving an early growth phase [17]. Abdel-Nour et al. add the complementary observation that *Legionella* adheres well to several plastics commonly used in plumbing, whereas copper inhibits attachment [18].

The practical implication is specific: gaskets, hoses and seals are not incidental components but high-productivity niches disproportionate to their surface area, and newly installed material is at its most supportive when new.

### 1.6. Gaps in the Existing Literature

Several reviews already survey *Legionella* ecology, detection and control. Lin, Stout and Yu appraised hospital disinfection modalities against a four-step framework [1]; Abdel-Nour et al. reviewed biofilm-associated persistence mechanisms [18]; Sciuto et al. surveyed disinfection and risk assessment in domestic systems [19]; Xi et al. systematically reviewed the factors governing chlorine-based disinfectant efficacy in premise plumbing [20]; Quon et al. meta-analysed log-reduction values for building-water interventions [21]; Ortiz et al. reviewed mechanistic modelling of *Legionella* in building water systems [22]; and Hammes et al. set out a research agenda for the coming decade [23]. These reviews are, for the most part, organised by domain—ecology, methods, control, and regulation—and evaluate the evidence within each domain. The present review is intended to complement rather than replace them.

Three gaps motivated the present review, each arising at the boundaries between those domains. First, the VBNC literature and the disinfection-efficacy literature are ordinarily reviewed separately, so the consequences of verifying culture-based interventions with a culture-based assay are rarely made explicit; Section 2.4 and Section 4 are therefore read jointly here. Second, existing reviews generally report what each modality achieves; this review additionally records the study design from which each claim derives and presents that appraisal in tabular form, so that design and conclusion can be weighed separately by the reader. Third, the mismatch between the units in which molecular methods report and the units in which regulatory thresholds are set has not, to the best of author’s knowledge, been treated as a single problem spanning metrology, epidemiology and regulation; Section 5.6 attempts this.

This is accordingly a critical narrative review with a declared interpretive position, not a systematic review. It does not attempt exhaustive coverage or quantitative synthesis, and where meta-analytic estimates are available, they are cited rather than recalculated [20,21]. Passages representing the author’s interpretation are identified as such, and alternative readings are noted where the evidence admits them.

### 1.7. Literature Search Strategy and Study Selection

Literature was identified by structured searching of PubMed/MEDLINE, Scopus, Web of Science and Google Scholar, supplemented by backward and forward citation tracking from retrieved records and by targeted retrieval of normative documents from ISO, WHO, ECDC, ASHRAE, CDC, CMS and the UK Health and Safety Executive. Searches were run during manuscript preparation and updated during revision.

Search terms combined a population term (“*Legionella*” OR “*Legionella pneumophila*” OR “Legionnaires’ disease”) with domain terms in four blocks: (i) ecology—“biofilm”, “amoeba”, “*Vermamoeba*”, “*Acanthamoeba*”, “premise plumbing”, and “building water system”; (ii) physiological state—“viable but non-culturable”, “VBNC”, “resuscitation”, and “infectivity”; (iii) detection—“culture”, “ISO 11731”, “qPCR”, “viability PCR”, “propidium monoazide”, “digital PCR”, “ddPCR”, “LAMP”, “metagenomic”, and “whole-genome sequencing”; and (iv) control and regulation—“disinfection”, “monochloramine”, “chlorine dioxide”, “copper-silver ionisation”, “ultraviolet”, “point-of-use filtration”, “thermal disinfection”, “water safety plan”, “water management program”, and “action level”.

Records were restricted to peer-reviewed publications in English. No lower date limit was applied because several load-bearing primary studies predate 2010 and remain the most detailed available on their specific questions. Conference abstracts, non-peer-reviewed reports and manufacturer literature were excluded.

Studies were selected for detailed appraisal on the basis of three criteria: (i) they reported primary quantitative data on *Legionella* in engineered water systems or provided systematic or meta-analytic synthesis of such data; (ii) they reported sufficient methodological detail for the study design to be evaluated—sampling frame, comparator, endpoint definition and detection method; and (iii) they bore on at least one of the three gaps set out in Section 1.6. Where several studies addressed the same question, priority was given to those with concurrent controls, multiple sites, longer follow-up, or explicit reporting of detection-method limitations. Studies cited principally for background are identified as such in the text.

Because this is a narrative rather than a systematic review, no PRISMA flow diagram is presented, and no formal risk-of-bias instrument was applied. In place of a formal instrument, the design features considered relevant to each claim are recorded explicitly in tabular form, so that the basis for the appraisal is visible to the reader. One limitation of this approach should be acknowledged: the emphasis on design quality may under-weight consistent field observations that were not obtained under controlled conditions.

## 2. Microbial Ecology and the Biofilm Niche

### 2.1. An Accidental Pathogen

*Legionella* is adapted to free-living amoebae, not to humans. The virulence apparatus permitting intracellular replication in *Acanthamoeba* is substantially the apparatus permitting replication in alveolar macrophages, and human infection is therefore a byproduct of amoebal parasitism [24,25,26]. Dietersdorfer et al. describe humans as accidental hosts [5]. Abdel-Nour et al. note that co-evolution with multiple protozoan species produced mechanisms allowing a very broad host range, including human cells [18].

The molecular basis is the Dot/Icm-type IVB secretion system, which translocates several hundred effector proteins into host cells—a repertoire whose catalogued size has grown with successive studies, so no fixed count is cited here—and is essential for intracellular replication [24,27].

Two points of critical importance follow, and they are frequently elided in the applied literature. First, selective pressure in engineered systems is exerted by protozoan predation and physicochemical stress, not by human immunity. Interventions evaluated solely against planktonic *Legionella* in axenic culture may not fully reflect conditions in complex environmental biofilms. Second, amoebal cysts protect intracellular bacteria from disinfectants [5]. Abdel-Nour et al. state the consequence directly: biofilms produced with *Legionella* in the presence of thermotolerant amoebae allow the organism to persist after heat treatment, demonstrating that amoebae provide a protective niche [18].

### 2.2. The Amoebal Replication Niche

Free-living amoebae—principally *Acanthamoeba* spp., *Vermamoeba vermiformis*, *Naegleria* spp. and *Willaertia* spp., together with ciliates such as *Tetrahymena*—constitute the primary replication niche [26,28]. Extracellular *Legionella* in oligotrophic water replicate poorly if at all; amplification to concentrations relevant to human infection requires intracellular multiplication.

Abdel-Nour et al. supply the quantitative link: the amount of *Legionella* in biofilms is directly correlated with protozoan biomass, and in vitro models show that the presence of amoeba species promotes *Legionella* biofilm formation [18]. They also note the organism can grow on debris from dead amoebae, so amoebae may encourage replication indirectly as well as by hosting it [18].

Margot et al. identified *V. vermiformis* as the dominant protist via 18S rRNA gene amplicon sequencing across their eight-week biofilm experiment, attributing successful *Legionella* colonisation substantially to its omnipresence [17]. Their proposed mechanism is testable: *V. vermiformis* grazes readily on young biofilm and thereby provides nutrients supporting intracellular growth [17].

The study was not designed to test this hypothesis directly. Amoebal and *Legionella* abundance co-varied; grazing was not directly observed, and the authors note that the resolution of their eukaryotic community analysis was likely limited by the small biofilm surface area studied [17]. The association is consistently observed, and the proposed mechanism is plausible, but this experiment does not establish a causal claim.

### 2.3. Biofilm as Structural Refuge: A Revised Interpretation

Biofilm is the default state of microbial life in premise plumbing. The extracellular polymeric substance matrix limits oxidant diffusion, impedes protozoan grazing and shear removal, and generates metabolic heterogeneity, producing slow-growing subpopulations intrinsically tolerant to disinfectants targeting active metabolism [18,29,30].

Abdel-Nour et al. supply direct evidence for the metabolic argument: *Legionella* grown on a solid surface is more resistant to killing by iodine than broth-grown bacteria, and sessile and planktonic cells in biofilms have different gene expression profiles [18]. Biofilm location also matters—sediment biofilms shield the organism from UV radiation [18].

Abdel-Nour et al., writing in 2013, framed the question as open: it remained unclear whether material-dependent differences in colonisation reflected direct surface–*Legionella* interactions or whether different plumbing materials select for different pioneering species that establish the initial biofilm, with *Legionella* colonising afterwards [18]. Margot et al. subsequently provided evidence for the first alternative. In their eight-week experiment on new EPDM material fed with non-chlorinated tap water, culturable *L. pneumophila* established rapidly—a median of 8.6 × 10^1^ MPN/cm^2^ after week 1, peaking at 3.1 × 10^4^ MPN/cm^2^ by week 4 [17]. Intact cell counts rose almost eightfold, from 1.2 × 10^5^ cells/cm^2^ at week 1 to 9.5 × 10^5^ cells/cm^2^ at week 5, then declined [17]. Their conclusion is explicit: pathogenic *Legionella* species can be among the earliest colonisers [17].

Two further findings bear directly on control:

- Co-occurring species behave differently. *L. rubrilucens* was an early coloniser, whereas *L. geestiana* was a late coloniser [17]. Genus-level monitoring conflates organisms with different colonisation dynamics.
- The biofilm is a source, not merely a refuge. Once *Legionella* established in the biofilm, suspended-phase concentrations remained high and stable, at approximately 10^4^ gene copies/mL [17]. This is the mechanistic basis for treating bulk-water sampling as an indirect and lagging indicator of a surface-attached reservoir.

Appraisal and limits. This is a single-material, single-water, laboratory-scale system at 37 °C with a 3.5-day residence time, seeded deliberately to 80 MPN/L [17]. The authors state that results cannot be generalised across building conditions while expecting the broad temporal dynamics to be inherent to biofilm maturation [17]. These data do not indicate that *Legionella* is invariably an early coloniser; they indicate that the assumption that it cannot be is not well supported, particularly for newly commissioned or recently renovated systems.

### 2.4. The Viable-but-Non-Culturable State

The applied and mechanistic literatures diverge most sharply on the viable-but-non-culturable state, which is central to the argument developed here. The evidence is set out first, and the interpretation placed on it is then stated separately. The core finding. Alleron et al. treated *L. pneumophila* biofilms with monochloramine and monitored culturability, viability and total DNA in parallel [31]. Doses ≥ 1 mg/L produced total loss of culturability, with no colonies recoverable for at least 145 days [31]. Over the same period, qPCR showed that total enumeration was essentially unchanged between treated and untreated populations—9.7 × 10^4^ GU/cm^2^ for the untreated population versus 4.3 × 10^4^ GU/cm^2^ after 10 mg/L monochloramine at day 20—while culturability differed absolutely (1.6 × 10^3^ CFU/cm^2^ for the untreated population versus zero in treated biofilm) [31]. BacLight staining showed approximately 29% of cells remained membrane-intact across all treated doses at 15 days, against 45% in untreated biofilm; at 145 days, roughly 10% retained esterase activity [31].

One interpretation consistent with these data is that a treatment producing a culture-negative result may have converted a culturable population into a persistent VBNC reservoir of comparable size so that culture records indicate an eradication while a substantial population persists. An alternative interpretation—that much of the residual genomic signal derives from cells damaged beyond recovery—cannot be excluded from these data alone and is considered below. Abdel-Nour et al. reached the same conclusion from an independent review of the literature: treatment of water systems with biocides can make *Legionella* enter the VBNC state, which makes accurate assessment of contamination levels cumbersome because it requires co-culturing with amoeba to lift that state [18].

Whether VBNC cells pose a genuine clinical risk remains unsettled, and the evidence reviewed here supports a matter of degree rather than an all-or-nothing answer. Dietersdorfer et al. [5] provide the strongest evidence identified in this review supporting the clinical relevance of the VBNC state. Six strains—four *L. pneumophila* SG1 strains, one SG6 strain, and one *L. micdadei* strain—were starved in ultrapure water at 45 °C for up to a year and co-cultured with *Acanthamoeba*, THP-1 macrophages, and primary human monocyte-derived macrophages [5]. Several strains directly infected primary human macrophages after more than 220 days of starvation, and some regained culturability after passage through primary macrophages [5].

The accompanying reduction in infection efficiency is an equally important component of that finding. Culturable controls infected approximately 61–65% of GM-CSF macrophages and 40–47% of M-CSF macrophages at MOI 50 [5]. After ≥221 days of starvation, the same strains infected 0.7–4.6% (GM-CSF) and 0.9–6.3% (M-CSF) at MOI 100, requiring at least three days in co-culture [5]. The authors calculated that between 700 and 6300 of 1 × 10^7^ starved VBNC cells remained infective, against 40,000–65,000 of 5 × 10^6^ culturable cells [5]—roughly a two-orders-of-magnitude reduction in infectious efficiency. Al-Bana et al., cited therein, reported only one to three of 100,000 starved VBNC cells resuscitating in amoebae [5].

Dietersdorfer et al. offer one of the more methodologically comprehensive studies on VBNC infectivity reviewed here [5]. Strengths of their experimental design include the testing of six distinct clinical and environmental strains, the use of primary human macrophages alongside cell lines, and evaluation of a year-long time course. However, several methodological limitations warrant consideration. Ultrapure water at 45 °C is a starvation model, not a plumbing system; MOIs of 100–400 are far above plausible environmental exposure, and the authors state explicitly that further quantitative research is needed to determine whether and how many starved VBNC cells can cause human disease [5]. The companion study on high-temperature-induced VBNC forms [32] should be read alongside it.

These data therefore suggest an intermediate position between the view that VBNC cells constitute an active reservoir of infection and the view that they represent biologically inert or non-viable populations. Under the examined conditions, VBNC *Legionella* retained infectivity at substantially reduced efficiency and persisted in numbers large enough that a two-orders-of-magnitude efficiency penalty does not, by itself, establish irrelevance. Whether the residual risk is material would depend on the ratio of VBNC to culturable cells in operating systems—a ratio that approached the entire population after disinfection in the laboratory biofilm of Alleron et al. [31], although the extent to which this holds in buildings is unknown. That ratio, rather than the infectivity of individual cells, is arguably the more informative quantity, and it is rarely reported. Readers who weight the efficiency penalty more heavily may reasonably regard the residual risk as small.

Alleron et al. found that amoebal resuscitation of monochloramine-treated *Legionella* succeeded at 1 mg/L but failed at higher doses, an effect they attribute speculatively to irreparable cell damage [31]. Dietersdorfer et al. note a second Alleron study in which monochloramine-triggered VBNC cells recovered neither infectivity nor culturability despite accumulating virulence-related proteins, including Mip [5]. Resuscitability is therefore dose- and stressor-dependent, and higher doses may produce genuinely dead cells rather than dormant ones. This finding presents a key nuance to the broader VBNC hypothesis and warrants careful consideration.

### 2.5. Resistance and Tolerance Mechanisms

Beyond amoebal and biofilm protection, *Legionella* possesses intrinsic stress-tolerance mechanisms: catalase-peroxidases and superoxide dismutases mitigating oxidative damage, RpoS and the LetA/LetS system coordinating transition to a stress-resistant transmissive phenotype, and metal efflux systems [25,26,33,34].

Abdel-Nour et al. note an unresolved confound: because of *Legionella*’s intracellular lifestyle within protozoa, it is difficult to determine whether its resistance in environmental biofilms stems from biofilm structure, amoebal association, or both [18]. This confound is rarely acknowledged in the applied disinfection literature, which frequently attributes treatment failure to biofilm penetration without fully accounting for the protozoal alternative—a distinction with direct implications for control strategy (Section 4.3).

Distinguishing heritable resistance from phenotypic tolerance is important but frequently conflated. Reports of resistant *Legionella* often describe tolerance conferred by physiological state or physical protection rather than genetic change. The distinction matters: tolerance is reversible and addressable by improving disinfectant delivery, whereas heritable resistance would demand a change of agent. Copper–silver ionisation is the clearest case. Reported treatment failures in German systems have been attributed to acquired resistance, but Lin et al. argue that these failures instead reflected ion concentrations held below the effective range, together with pH and competing-ion effects on ionic copper availability, rather than any heritable change in the organism [35]. On the basis of evidence located for this review, no study has demonstrated stable, heritable copper or silver resistance in *Legionella* arising under field ionisation, and the tolerance-selection hypothesis should therefore be reported as unresolved rather than established.

## 3. Next-Generation Detection Technologies

### 3.1. Culture: ISO 11731 and the Persistence of the Reference Method

ISO 11731 remains the internationally recognised reference method and the basis of most regulatory action levels [36]. Its strengths are substantial: unambiguous evidence of culturable organisms and recovery of isolates for serogrouping and sequence-based typing, which is irreplaceable in outbreak attribution. Bai et al. record its clinical performance as 60–80% sensitivity with nearly 100% specificity and note that it identifies all known *Legionella* species and serotypes—a capability no targeted molecular assay matches [6].

Its limitations are also substantial and, in the VBNC context, materially constrain its use for verification. Turnaround is 7–14 days. Lu et al. describe the standard environmental workflow—200 mL filtered through a 0.44 μm membrane, resuspension, acid pretreatment, and GVPC plating—noting that acid treatment precedes plating specifically to suppress competing flora [37]. While necessary for the suppression of overgrowth, selective acid pre-treatment can also reduce the overall recovery of target *Legionella*. Falzone et al. add that culture is impractical where growth is inhibited by accompanying bacteria [38], and Tiwari et al. note that VBNC organisms induce systematic underestimation [39].

### 3.2. Quantitative PCR and the Interpretive Problem

qPCR targeting mip, 16S rRNA regions, or serogroup-specific markers delivers results within hours, with ISO/TS 12869 providing a standardised framework [40,41]. Bai et al. report clinical sensitivity of 17–100% and specificity of 95–100%, explicitly listing widely fluctuating sensitivity as a limitation [6].

Alleron et al. state the environmental interpretive problem directly. Reflecting on their own data, they note that qPCR gave similar values across all analysed samples—between 10^4^ and 10^5^ bacteria equivalents/cm^2^—while parallel culture showed some of those same samples contained no culturable cells and over 50% dead cells [31]. They concede they cannot determine whether their qPCR protocol distinguishes dead from live cells while maintaining they succeeded in enumerating VBNC bacteria [31].

These observations illustrate the interpretive difficulty compactly: qPCR detects a population that culture cannot see, culture distinguishes a physiological state that qPCR cannot resolve, and neither method alone answers the question facing a building operator. Viability PCR partially bridges the gap but does not close it, and dye penetration into biofilm aggregates remains imperfect [42,43].

### 3.3. Digital Droplet PCR: Documented Advantages and Their Limits

ddPCR partitions a bulk reaction into thousands of picolitre droplets, scores each as positive or negative at the endpoint, and estimates the concentration via Poisson statistics [39]. Three studies bear directly on its performance for *Legionella*, and together, they support a narrower claim than some primary reports have been taken to imply.

#### 3.3.1. The Primary *Legionella* Comparison

Falzone et al. compared ddPCR against RT-qPCR using serial dilutions of *L. pneumophila* serogroup 1 from 10^7^ down to 10 CFU/mL, as well as in an in vitro heat-shock model [38]. Both methods detected the legally relevant concentration of 10 CFU/mL, but RT-qPCR did so only at a very late C_t_ of 31.10, whereas ddPCR returned a quantifiable 0.29 copies/μL [38]. Linear regression across the dilution series, excluding the saturating 10^7^ CFU/mL point, gave r^2^ = 0.8388 for ddPCR against r^2^ = 0.5228 for RT-qPCR [38].

The heat-shock result is the more consequential one, and it connects directly to Section 2.4. After 80 °C treatment for 30 min over three consecutive days, RT-qPCR returned a false-positive signal at day 5 post shock (C_t_ 35.18), which the authors attribute to cell debris and residual degraded DNA producing nonspecific amplification [38]. ddPCR scored all heat-shocked samples negative while correctly tracking growth in untreated flasks from 1.9 to 5.3 copies/μL over three days and to 25.5 copies/μL by day five—an increase RT-qPCR failed to resolve [38].

Appraisal and a limitation the authors themselves state. This is an in vitro simulation using a single organism in sterile water. Falzone et al. acknowledge that environmental and clinical samples contain many other bacteria that, with cellular debris and degraded DNA, interfere with detection [38]. The study demonstrates ddPCR’s resilience to fragmented DNA under conditions specifically constructed to isolate that variable—it does not demonstrate performance in real premise plumbing matrices.

A caution on transferring the heat-shock finding. Falzone et al. might be read as showing that ddPCR can verify thermal disinfection where qPCR cannot; this reading extends beyond what the data support. Their 80 °C protocol was designed to kill, and ddPCR scored the samples negative—consistent with killing. It does not follow that ddPCR distinguishes dead from VBNC cells. Like all nucleic acid methods, ddPCR quantifies genomes irrespective of viability [39]. What Falzone et al. demonstrate is that ddPCR is less prone than qPCR to false positives arising from degraded DNA—a useful property for post-treatment verification but distinct from viability discrimination.

#### 3.3.2. Reference Material and Standardisation

Baume et al. applied ddPCR to the *Legionella* DNA Certified Reference Material, qualifying linearity, limit of detection and limit of quantification against ISO/TS 12869 criteria [44]. Linearity exactitude was ≤0.15 log_10_ from 42,000 down to 15 GU/reaction; LOD was validated at 2.5 GU/reaction in duplicate and 5 GU/reaction in simplicate; LOQ was validated at 15 GU/reaction in duplicate and 25 in simplicate [44]. Estimating the CRM value across 10 tubes and 71 replicates gave 1782.3 ± 144.5 GU/5 μL, equating to 9,089,730 ± 736,950 GU per tube, against the 2009 certified value of 10,627,646 ± 1,631,787—not significantly different (*p* = 0.065) [44].

The significance of this result relative to the sensitivity claims. The principal argument for ddPCR is arguably not that it detects more but that it quantifies without a calibration curve. Baume et al. make the consequence concrete: prior qPCR-based stability monitoring depended on the CRM itself for calibration, so an observed variation could not be attributed with certainty to DNA degradation rather than qPCR uncertainty [44]. ddPCR breaks that circularity. Given that a central concern of this review is circular validation (Section 4.4), a method that removes circularity from the measurement itself is of particular relevance.

Two features require disclosure. Four of the seven authors are Bio-Rad employees, and the platform used was Bio-Rad’s QX200 [44]. More consequentially for reproducibility, the assay target is identified only as a proprietary *L. pneumophila*-specific gene, with primer and probe sequences marked as Bio-Rad confidential information available on request [44]. An independent laboratory cannot reproduce this work from the paper alone—a real limitation on a study whose stated purpose is standardisation.

#### 3.3.3. What the Broader Water-Microbiology Literature Adds

Tiwari et al. reviewed 63 publications comparing dPCR and qPCR or applying dPCR in health-related water microbiology [39]. Their synthesis supports the qualified reading advanced here.

- Sensitivity and precision generally favour dPCR but not universally. Most studies have reported greater analytical sensitivity for dPCR, including that by Falzone et al. for *Legionella* specifically [39]. But at least two have reported qPCR as more sensitive, and Wang et al. found mixed inhibition results, with qPCR more resistant to humic acid and dPCR more resistant to calcium [39].
- The dynamic range is worse, not better. qPCR spans up to 7–8 log_10_; dPCR is constrained to roughly 3–4 log_10_ [39]. Samples above range must be diluted and rerun. Falzone et al. encountered exactly this: their 10^7^ CFU/mL point saturated the system and was excluded from regression [38].
- Cost may be prohibitive for routine monitoring. Tiwari et al. conclude that as currently configured, dPCR is likely cost-prohibitive for widescale adoption for routine microbial water quality monitoring though justifiable for research or for validating qPCR control materials [39].
- Platform comparisons are immature. Most published water-microbiology dPCR work has used Bio-Rad QX100/QX200 (Bio-Rad Laboratories, Hercules, CA, USA) systems—used in [39] and [9] of the studies reviewed, respectively—with other platforms sparsely represented. Both *Legionella* studies appraised here used Bio-Rad platforms [38,44].

The decisive limitation stated by the reviewers themselves. Tiwari et al. close by noting that, like all molecular methods and of particular importance for public-health water microbiology, dPCR cannot distinguish viable from nonviable infectious agents, so extrapolation from gene-copy measurements to health risk remains tenuous [39].

ddPCR is a better quantification technology than qPCR on most axes that matter for post-treatment verification: no calibration curve, greater precision at low concentrations, and resilience to inhibitors and degraded DNA. It is not a solution to the VBNC problem. Coupling viability pretreatment with droplet digital quantification is a logical next step, and the pretreatment half of that pairing is already mature: Lee and Han optimised PMA-qPCR for *Legionella* across the tap-water supply chain, establishing 100 µM PMA as the concentration giving the greatest live–dead separation without cytotoxicity to viable cells and a 386 bp amplicon as the target length that excluded dead-cell signals without loss of amplification efficiency [45]. They also defined the method’s operational envelope, showing reliable live–dead discrimination only in samples below 10 NTU, above which particle shading impairs dye penetration and photoactivation [45]. What has not been evaluated on the basis of the evidence located for this review is the transfer of those optimised parameters onto a droplet digital platform for *Legionella* in premise plumbing, where turbidity, biofilm-derived aggregates and low target concentrations differ materially from the distribution-system matrices in which PMA-qPCR was validated. The gap is therefore the platform pairing and its validation in building water, not viability pretreatment as such.

### 3.4. Rapid On-Site Biosensors and Field Platforms

Lu et al. developed a real-time LAMP platform targeting 16S rRNA, with LS-LAMP for genus-level detection and LP-LAMP discriminating *L. pneumophila* [37]. Against 61 reference strains, specificity was 100% for both assays, with sensitivity for *L. pneumophila* between 52 and 5.2 copies per reaction [37]. Across 107 environmental water samples from cooling towers, fountains and artificial lakes, sensitivity was 100% for both assays (51/51 and 18/18), while specificity fell to 91.6% and 93.3% respectively [37]. Amplification proceeds isothermally at 60–65 °C, requiring no thermal cycler, with detection inside two hours [37].

The instrumentation advantage is substantive: eliminating thermal cycling is what makes field deployment plausible, and sensitivity is competitive with laboratory qPCR. Three limitations qualify these results. First, the drop from 100% specificity against reference strains to 91.6% against environmental samples is the operationally relevant figure—roughly one in twelve genus-level positives was a false positive against culture and serology as reference, though when culture is the comparator, “false positive” is ambiguous in exactly the way Section 3.2 describes. Second, the samples were of cooling towers and surface waters, not potable premise plumbing, where target concentrations are lower and the concentration step becomes limiting. Third, this is a single-laboratory development and validation study. Bai et al., reviewing clinical application, reach a consistent assessment: LAMP is rapid and low-budget with specificity above 90% but is not promoted in clinical practice and lacks adequate research [6].

The dominant technical barrier across all field platforms remains sample concentration. Detecting culture-relevant concentrations in a small field-processed volume requires the concentration of litres of water, and robust field-deployable concentration remains the rate-limiting step.

### 3.5. Comparative Assessment of Detection Technologies

Table 1 summarises detection technologies against operationally relevant criteria, with an explicit column for evidence quality, which comparisons of this kind do not usually include.

### 3.6. Toward an Integrated Monitoring Architecture

The evidence does not support replacing culture with molecular methods or retaining culture alone. A defensible architecture is tiered. Tier 1, continuous or high-frequency: online physicochemical monitoring (temperature, disinfectant residual, flow, ORP, and pH) supplemented, where feasible, by flow cytometry. These are the operational control points and can be monitored at a temporal density microbiological sampling cannot approach. Tier 2, routine screening: qPCR or viability-qPCR at defined intervals, trended over time; the value lies in the trend and spatial pattern, not any individual result. Tier 3, confirmation: ddPCR for precise quantification at low concentrations [38,44], with parallel culture where isolate recovery is required. Tier 4, epidemiological investigation: culture with sequence-based typing or whole-genome sequencing for clinical–environmental matching—a function irreplaceable by molecular quantification alone.

## 4. Advanced Engineering Controls and Disinfection

Conventional treatments of this topic summarise what each method achieves. The approach taken here is complementary rather than substitutive: it also asks what study designs the reported claims rest on, since in this literature, design and conclusion are closely coupled. Two recent syntheses allow that appraisal to be set against quantitative evidence: Xi et al. systematically reviewed 117 studies of chlorine-based disinfection in premise plumbing and identified 26 operational, evolving and stable factors modifying efficacy [20], while Quon et al. meta-analysed log-reduction values across 45 building-water intervention studies [21]. Where their conclusions bear on the appraisal below, they are cited alongside it.

### 4.1. The Primacy of Thermal and Hydraulic Management

When applied to hydraulically or thermally compromised systems, chemical disinfection primarily serves as a supplemental measure, which can constrain its overall long-term efficacy. Maintaining hot-water storage above approximately 60 °C and return-loop temperatures above approximately 51 °C, with cold water below approximately 20 °C, suppresses growth thermodynamically [1,46]. The practical obstacles are scald prevention, energy cost, heat gain to cold-water lines, and hydraulic imbalance producing cool branches. Thermal balancing of recirculation systems—verified by measurement at return points, not assumed from design—is among the highest-yield interventions available.

Marchesi et al. provide field confirmation from the point-of-use direction: electric boilers serving one or two adjacent rooms were rarely contaminated (5/57 samples), and all five positives occurred where water temperature was 40–42 °C, with a single exception at 57.8 °C [47]. Their operational threshold is explicit—boiler temperatures below 58 °C are ineffective [47].

Stagnation management is the complementary control. Systematic flushing of low-use outlets, elimination of dead legs, right-sizing of service lines, and documented commissioning of newly constructed or long-vacant buildings all matter. Marchesi et al. supply an unusually clean natural experiment: of three buildings constructed in the 1990s with identical galvanised-steel hot-water networks and identical construction methods, two were never contaminated and one was heavily colonised [47]. The only difference the authors could identify was that the two uncontaminated buildings were constructed rapidly and occupied immediately after testing, whereas in the colonised building, occupation was progressive, with sections of the water system remaining unused for long periods after testing [47]. Their recommendation follows directly: for new structures, either drain the system completely until use or put it into service immediately. Rhoads et al. add a complementary caution from the operational side: the response of *Legionella* to precautionary flushing after periods of low occupancy varied substantially between buildings, so flushing protocols validated in one system should not be assumed to transfer to another [48].

### 4.2. Secondary Disinfection Options

- Monochloramine. Generated on site, monochloramine is a weak but persistent oxidant with low reactivity toward organic matter, giving exceptional residual stability and biofilm penetration [49,50,51]. Field evidence from healthcare facilities supports substantial reduction. Farina et al. report ten years of monochloramine disinfection combined with a water safety plan and extensive environmental sampling in an Italian hospital, with sustained control of *Legionella* and other waterborne pathogens [52]; as an uncontrolled single-site programme, it shares the design limitations discussed in Section 4.5, but it extends the field record for monochloramine by a further decade. Principal concerns are nitrification, nitrogenous disinfection byproducts including NDMA, incompatibility with dialysis, potential lead release, and the operational demands of chlorine-to-ammonia ratio control. Abdel-Nour et al. note the important qualification that chloramine, among the most potent chlorine-derivative biocides, does not completely eradicate *Legionella* from aquatic biofilms [18]—a statement whose mechanism Alleron et al. supply directly (Section 2.4).
- Chlorine dioxide. Chlorine dioxide is a selective oxidant that is effective across a broad pH range with good biofilm penetration and no trihalomethane formation [1,53]. Its residual behaviour in hot water is the contested question addressed in Section 4.3.
- Copper–silver ionisation. Electrolytic release of Cu^2+^ and Ag^+^ ions provided residual protection to distal outlets, including within biofilm [2,54,55]. Efficacy is strongly pH-dependent, declining above approximately pH 8 as silver precipitates [35]. Electrode fouling requires routine maintenance, and ion concentrations must be verified analytically rather than assumed from current settings.
- Ultraviolet irradiation. UV-C inactivates by nucleic acid damage, with no chemical residual and no byproduct formation. Its defining limitation is the absence of a residual: UV protects only water passing through the reactor and confers no protection against downstream biofilm, which is where premise-plumbing *Legionella* predominantly resides [1]. Abdel-Nour et al. add that chlorine derivatives are more efficacious than UV for disinfection of *Legionella* and that sediment biofilms provide protection from UV radiation [18].
- Point-of-use filtration. Membrane filters (typically 0.2 μm) provide an absolute physical barrier at the exposure interface—the most reliable available protection for defined high-risk populations [1,56,57]. Marchesi et al. observed no contamination whatsoever at filtered outlets [47]. Limitations are finite service life, replacement cost, and the fact that filtration addresses exposure without addressing system colonisation.

### 4.3. Chlorine Dioxide in Hot Water: Reconciling Divergent Reports

Chlorine dioxide is frequently described as decaying rapidly at hot-water temperatures, with the implication that its utility in hot systems is limited. The evidence set out below indicates that this description requires qualification.

In a 765-bed Italian university hospital, continuous chlorine dioxide was installed in 2005 in hot water plants A and B, targeting 0.3 ppm at distal outlets [47]. Over a three-year observation period, it maintained *Legionella* at low levels, with a strong negative correlation between chlorine dioxide concentration and *Legionella* concentration (r = −0.70, *p* < 0.01) [47]. The regression permitted a quantitative dose recommendation: 0.3 ppm associated with <100 cfu/L and 0.6 ppm with <25 cfu/L, the detection limit [47]. Positivity fell from 65/67 (97.0%) pre-treatment to 43/80 (53.7%) post installation (*p* < 0.001) [47].

Lin, Stout and Yu, by contrast, emphasise low hot-water residuals, reporting studies where hot-water chlorine dioxide fell to 0.04–0.08 mg/L against 0.3–0.68 mg/L in cold water, and conclude that chlorine dioxide does not fulfil their four criteria [1].

Reconciling the two accounts. These findings are not necessarily irreconcilable. Marchesi et al. dosed to achieve 0.3 ppm at distal outlets rather than at the source and monitored to confirm it [47]. The studies Lin et al. cite dosed at source and measured decay downstream [1]. On this reading, the divergence concerns dosing strategy and verification at least as much as chlorine dioxide chemistry; it is an interpretation, and the two bodies of work were not designed to be compared. It gains some support from Xi et al., whose systematic review ranks typical field efficacy as monochloramine > chlorine dioxide > free chlorine while identifying delivered concentration, temperature, stagnation, biofilm and protozoa among 26 factors that modify that ranking [20]. That reading is supported by Marchesi et al.’s own caution: one or two days of inadequate levels suffice for recontamination, and strict injection control is required [47].

Two findings from Marchesi et al. [47] complicate their own recommendation:

- Serogroup selection: After chlorine dioxide, *L. pneumophila* serogroup 1 was isolated more frequently: 16/43 (37.2%) versus 5/65 (7.7%) pre-treatment (*p* < 0.001) [47]. Total counts fell while the proportion of the most clinically significant serogroup rose. The authors note no clinical cases resulted, and this is why they began testing monochloramine [47]. Total *Legionella* concentration and clinical risk are not interchangeable endpoints, and a treatment can improve one while worsening the other.
- The protozoal explanation. The authors attribute rapid rebound after brief dosing failures to chlorine dioxide killing bacteria in the water stream but not those inside protozoa [47]—connecting directly to the confound Abdel-Nour et al. identify in Section 2.5 [18].

### 4.4. The Validation Framework and Its Circularity

Stout and Yu proposed a four-step framework for evaluating disinfection modalities: (1) demonstrated in vitro efficacy, (2) anecdotal reports from individual hospitals, (3) controlled studies of prolonged duration in individual hospitals, and (4) confirmatory reports from multiple hospitals over prolonged follow-up [2]. They then concluded, in the same paper, that copper–silver ionisation was the only disinfection modality found to satisfy all four evaluation criteria [2].

The paper supplying step 4 was the paper proposing the framework. Lin, Stout and Yu subsequently reproduced the framework in their 2011 review, citing Stout and Yu 2003 as its source, and concluded, again, that copper–silver ionisation is the only technology validated by the four-step criteria they recommend [1].

This framework design presents methodological considerations: because the multi-hospital survey fulfilling the fourth criterion was conducted by the framework’s originators, competing disinfection modalities faced an evaluation benchmark that was not systematically applied across all technologies. The framework is not unreasonable in content—the stepwise logic from laboratory to multi-site field validation is sound—but its application here does not constitute independent validation.

In fairness, three points must be recorded. Stout and Yu explicitly declare no financial interest in ionisation manufacturers; state they are not officers, board members, stockholders or paid consultants; and note the survey received no company support and no company input into design or conduct [2]. They also argue, with justification, that randomised comparative trials are impractical here, citing the diversity of hospital water systems, environmental variability, the infeasibility of a control group in an urgent situation, and the absence of an established standard for comparison [2]. They also acknowledge that conditions across the two survey periods may not have been comparable [2].

The impracticality argument is well founded, and no criticism of the authors is intended for not conducting a trial that could not feasibly have been conducted. The observation made here is narrower: where design constraints are unavoidable, correspondingly cautious language is warranted, and definitive claims regarding single modalities fulfilling all validation criteria should be interpreted with appropriate methodological caution. Readers who weight the consistency of the reported field outcomes more heavily may reasonably assess this differently.

### 4.5. Appraisal of the Copper–Silver Evidence Base

The 16-hospital survey [2] was based on mailed questionnaires sent to the first 16 US hospitals installing ionisation in 1995 and 2000, to which all 16 responded, covering systems with a mean of 435 beds operational for 5–11 years by 2000, and found that before installation 47% (7/15) reported more than 30% of distal sites positive [2]. After installation, 50% reported 0% positivity in 1995, and 43% still reported 0% in 2000 [2]. All 16 had experienced hospital-acquired Legionnaires’ disease before installation; 94% (15/16) reported none afterwards, and the single hospital reporting a case had none from 1995 to 2002 [2].

The reported outcome is substantial and should not be dismissed. The design nonetheless carries limitations that qualify its interpretation:

- No concurrent controls: Before–after comparison across a decade in which awareness, testing practice and infection control generally changed considerably.
- Self-reported outcomes in coarse bands: Positivity is recorded as 0%, ≤30%, or >30% [2]. Investigators did not sample. Sampling frequency varied from quarterly to annual [2].
- Culture-based endpoints throughout, so on the basis of the Alleron argument, incapable of distinguishing eradication from VBNC conversion [31].
- Ascertainment of the primary outcome is hospital-dependent: “No cases of hospital-acquired Legionnaires’ disease” depends on how hard each hospital looked, and diagnostic intensity plausibly co-varies with having installed a disinfection system—a concern sharpened by the diagnostic performance figures in Section 3.1 [6].
- Selection: These are the first 16 adopters—early, motivated institutions, 75% of which had already failed with other methods [2].

A stronger study in the same programme [55]. Liu et al. installed a single ionisation unit sequentially on two hospital buildings, using a third building on the same water supply (also colonised) as a concurrent control [55]. Distal-site positivity fell to zero in the first test building four weeks after activation and in the second after twelve weeks, while the control building remained positive throughout [55]. Recolonisation did not occur for 6–12 and 8–12 weeks after inactivation, and copper concentration was significantly higher in biofilm than in water—which the authors advance as the basis for the residual effect [55].

This is a materially better design than the survey, and the biofilm-accumulation finding supplies a mechanism for the observed lag. It is nonetheless a single 541-bed psychiatric hospital with three buildings, culture-based endpoints, and no clinical outcome. It supports the environmental claim well and does not address the disease-prevention claim. On the appraisal offered here, it warrants at least as much weight as the 16-hospital survey, although it is cited less frequently.

### 4.6. Shock Treatments: Consistent Rebound Across Independent Studies

Otherwise divergent sources converge on this point, which makes it among the better-supported conclusions in Section 4.

Marchesi et al. report that shock disinfections returned to pre-treatment contamination within one or two months [47]. Superheating (two days > 60 °C at distal points, eight times over four years) produced a non-significant reduction within the first month (2500 vs. 8100 cfu/L), after which values returned to baseline (7000 cfu/L) [47]. Shock hyperchlorination (20–50 ppm free chlorine at distal points for 1–2 h, twelve times over seven years) was initially effective but returned to or exceeded pre-treatment levels after two months [47]. Superheating actually increased the proportion of positive points (+30.5%) in their cost-effectiveness comparison [47].

Lin, Stout and Yu concur from an independent evidence base, describing hyperchlorination as among the least reliable and most costly modalities, noting that most of the 17 hospitals in their earlier review had since converted away from it [1]. Stout and Yu note that thermal eradication, a method their own group developed, is efficacious but that recontamination often recurs within months [2]. The meta-analysis of Quon et al. is consistent with this pattern: log-reduction values for responsive interventions such as heat shock and shock chlorination were generally smaller and more variable than those reported for sustained controls, and intervention performance depended strongly on incoming water quality and contextual conditions [21].

The VBNC connection and how to test it. The conventional explanation for rebound is incomplete biofilm penetration. Alleron’s data supply an additional mechanism: treatment may convert culturable cells to VBNC, which culture then scores as eradication, with subsequent apparent recolonisation partly reflecting resuscitation of a population that never left [31]. Abdel-Nour et al. note a third possibility that is rarely separated from the first two—that protection derives from the intracellular protozoal niche rather than from biofilm structure and that the two cannot easily be disentangled [18].

Falzone et al. make the required experiment concrete. Their heat-shock model showed qPCR generating a false positive from degraded DNA where ddPCR did not [38]. A field study monitoring a shock-disinfection cycle would therefore need three parallel measurements—culture, ddPCR, and viability-corrected molecular measurements—to separate three distinct populations: culturable, VBNC-intact, and dead-but-detectable populations. Neither culture-plus-qPCR nor culture-plus-ddPCR alone can do this. This review could not locate such a study.

### 4.7. Cost-Effectiveness: A Frequently Underreported Dimension

The work of Marchesi et al. is unusual in reporting costs alongside efficacy, and the figures reframe the discussion (Table 2). Their effectiveness ranking was filters > boilers > chlorine dioxide > hyperchlorination > thermal shock; the cost ranking was almost exactly inverted [47]. Filters achieved 100% negative samples but at a cost the authors calculate would reach approximately €1 million per year to serve their hospital’s 1000-plus outlets [47]. Their recommendation—chlorine dioxide throughout, with electric boilers for high-risk patients, at roughly €70,000 per year—is a cost-effectiveness judgement, not an efficacy judgement [47].

This framing appears less frequently in the US clinical literature. The relevant question is arguably not which modality is most effective in isolation but which combination achieves acceptable risk at sustainable cost—an answer that is necessarily building-specific. The same logic applies to detection: Tiwari et al. reach a structurally identical conclusion about dPCR—that its performance advantages may not justify its cost for routine monitoring [39].

### 4.8. A Null Result and Its Interpretation

Marchesi et al. detected no cases of hospital-acquired legionellosis across the entire ten-year period, despite persistent contamination [47]. They are appropriately candid that this prevents them from evaluating any procedure’s effect on infection risk [47].

They offer two explanations, and the first is the more interesting: their system was dominated by *L. pneumophila* serogroups 9 and 6, which are less frequently associated with disease than serogroup 1 [47]. The second is that their targeted controls for high-risk patients—boilers and filters—worked [47].

One implication of this observation is broad. If serogroup distribution substantially determines clinical risk, then total *Legionella* concentration—the quantity for which guidelines set thresholds and that disinfection studies most often report—may be an imperfect proxy for clinical outcome. This inference rests on a single site at which no cases were observed and is better treated as a hypothesis requiring multi-site testing than as an established relationship. Marchesi et al.’s own serogroup 1 selection finding [47] sharpens this considerably: their chosen treatment reduced the regulated quantity while enriching the dangerous one. The ascertainment problem compounds it: Marchesi et al. acknowledge that routine EIA urinary testing could have missed non-serogroup-1 infections [47], which is precisely the limitation Bai et al. quantify [6].

Table 3 summarises the secondary disinfection approaches discussed above. Each is graded by the design quality of the best evidence located for it, and what that evidence supports is separated from what it does not so that the two can be assessed independently.

## 5. Regulatory Frameworks and Public Health Management

### 5.1. The Paradigm Shift to Water Management Programs

The defining regulatory development of the past two decades is the transition from a sampling-and-threshold model to a proactive, hazard-analysis-based management model. Conceptually derived from HACCP and formalised in the WHO Water Safety Plan framework [58,59,60], this approach requires building owners to identify hazardous conditions, define control measures and control limits, monitor those limits, specify corrective actions, and verify programme effectiveness—rather than periodically sampling and reacting to exceedances.

The rationale is straightforward: *Legionella* concentrations in premise plumbing are spatially and temporally heterogeneous to a degree that makes any feasible grab-sampling regime a weak estimator of the true system state [7,61]. Managing the conditions that permit growth is more tractable and more preventive than attempting to measure the outcome.

### 5.2. ASHRAE Standard 188 and Guideline 12

ANSI/ASHRAE Standard 188, Legionellosis: Risk Management for Building Water Systems, first published in 2015 and revised in 2018 and 2021 (the 2021 edition is cited here), is the principal normative document in the United States [46]. It establishes minimum requirements for developing and implementing building water management programmes: the formation of a multidisciplinary programme team; description of the water systems with process flow diagrams from service entrance to points of use; identification of control locations and establishment of control limits; monitoring procedures, corrective actions, and confirmation that the programme is implemented as designed and is effective; and documentation with periodic review. ASHRAE Guideline 12 provides supplementary technical guidance [62].

Two features attract persistent criticism. First, Standard 188 is deliberately process-oriented and does not prescribe *Legionella* sampling frequencies or numeric action levels, leaving these to the programme team. Proponents argue this is appropriate, given system heterogeneity; critics note it may allow programs to achieve administrative compliance without necessarily demonstrating measurable microbiological reduction [61,63]. Second, as a voluntary consensus standard, it carries force only where adopted by reference into codes, regulation, contract, or accreditation requirements.

### 5.3. The CDC Toolkit

The CDC’s Developing a Water Management Program to Reduce *Legionella* Growth and Spread in Buildings translates Standard 188 into operational guidance [64]. It provides a structured, worksheet-based process for team formation, system description, hazard identification, control-measure definition, monitoring design, corrective-action planning, and verification and validation. Its practical contribution has been to lower the implementation barrier for facilities without in-house water treatment expertise. It remains guidance rather than regulation.

### 5.4. CMS Requirements

The Centers for Medicare & Medicaid Services memorandum QSO-17-30-Hospitals/CAHs/NHs, issued 6 June 2017 and revised 6 July 2018, which remains in force at the time of writing, represents the most consequential enforcement mechanism in the United States because it links compliance to participation in federal reimbursement programmes [65]. CMS expects hospitals, critical-access hospitals, and long-term care facilities to have water management policies and procedures to reduce the risk of the growth and spread of *Legionella* and other opportunistic pathogens. Facilities must conduct a facility risk assessment; implement a water management programme that considers ASHRAE Standard 188 and the CDC toolkit and includes control measures such as physical controls, temperature management, disinfectant-level control, visual inspections, and environmental testing; and specify testing protocols and acceptable ranges, documenting results and corrective actions [65]. The 2018 revision clarified that environmental testing for pathogens is at the discretion of the facility.

The mechanism converted a voluntary standard into a de facto requirement for a large segment of the highest-risk building stock. Its limitations mirror those of Standard 188: surveyors assess programme existence and documentation more readily than programme efficacy, and no numeric environmental thresholds are federally specified. The Joint Commission imposes a parallel accreditation requirement, and some state health departments and the Veterans Health Administration have promulgated more prescriptive requirements.

### 5.5. International Frameworks

- WHO. The WHO framework, articulated in the Guidelines for Drinking-water Quality [59], the dedicated *Legionella* and the Prevention of Legionellosis volume [58], and the Water Safety Plan Manual [60], provides the international conceptual foundation: system assessment, operational monitoring of control measures, management and communication procedures, and independent surveillance.
- European Union. The recast Drinking Water Directive (EU) 2020/2184 introduced an explicit risk-based approach extending to domestic distribution systems in priority premises, with *Legionella* named as a parameter for monitoring where risk assessment indicates [66]. This represents a significant extension of regulatory attention beyond the point of delivery into premise plumbing, historically a jurisdictional gap. The ECDC coordinates European surveillance, including the scheme for travel-associated legionellosis, an important mechanism for detecting clusters invisible to any single national system [67].
- United Kingdom. The Health and Safety Executive’s Approved Code of Practice L8 and the accompanying HSG274 technical guidance impose duties under health and safety legislation on those in control of premises, with defined expectations for risk assessment, competent responsible persons, monitoring regimes, and record keeping [68,69]. The UK framework is notably more prescriptive than the US model and has a longer enforcement history.
- Continental Europe. France, Germany, Italy and the Netherlands have among the longest-standing prescriptive regimes, with mandated monitoring frequencies and numeric action levels for defined building categories, together with mandatory reporting of exceedances [70,71,72]. Marchesi et al. illustrate the operational application: Italian guidelines specify no intervention below 100 cfu/L, clinical surveillance between 100 and 10^4^ cfu/L, and mandatory disinfection above 10^4^ cfu/L [47]. Falzone et al. describe a comparable regime, noting a limit of 10^2^ CFU/L, with total absence of *L. pneumophila* mandatory in units housing immunosuppressed patients [38].

### 5.6. Persistent Regulatory Gaps

- Absence of validated molecular action levels: Regulatory thresholds are expressed in CFU/L, yet the most operationally useful methods report genomic units per litre. No authoritative conversion exists, and the ratio is system-specific and highly variable [42]. Given Alleron’s demonstration that monochloramine can drive culture counts to zero while total genomic units remain at 10^4^–10^5^ GU/cm^2^ [31], a system can move from “mandatory disinfection” to “no intervention required” without the organism’s abundance changing materially. This represents a key point of divergence between current analytical capabilities and established regulatory frameworks.
- The metrological groundwork exists; the epidemiological work does not. ISO/TS 12869 provides a qPCR framework [41], and Baume et al. demonstrate that a certified reference material can be quantified by ddPCR against its criteria [44]. What is missing is the work connecting GU/L to human disease incidence that would justify setting thresholds in those units—and, as Section 1.1 shows, the surveillance data against which such a correlation would be established are, themselves, shaped by a serogroup-1-specific ascertainment method [6].
- Weak validation requirements: Programmes are commonly assessed on the basis of documentation rather than demonstrated microbiological control; independent third-party validation is rare [63].
- Sampling-design underspecification: Marchesi et al. note that Italian guidelines offered no advice on the frequency or number of sites to sample in non-epidemic situations, obliging them to design their own strategy—at least one remote point per 50 beds [47]. Verification frequency is similarly unsettled: The WHO recommends quarterly cultures for hospitals using systemic disinfection, while Allegheny County recommends annual culturing of high-risk units and Maryland recommends flexibility with quarterly culturing after an outbreak [1].
- Fragmented jurisdiction: Responsibility divides among drinking-water regulators (to the property line), building and plumbing codes (design), occupational safety authorities (workplace exposure), and health departments (outbreak response), with premise plumbing frequently falling between them [7].
- Under-regulation of non-healthcare settings: Multi-unit residential buildings, hotels, and long-term care facilities collectively account for a substantial disease burden with far less regulatory attention than acute-care hospitals [71,73]. Recent surveys extend the point beyond these building classes: Nielsen et al. detected *Legionella* in primary-school hot water systems in two Danish municipalities, a building category subject to minimal routine surveillance in most jurisdictions [74].
- Cooling-tower registration: Registries enabling rapid identification of aerosol sources during outbreak investigation exist in some jurisdictions and not others, materially affecting investigation speed [75].

## 6. Emerging Frontiers, Research Gaps, and Future Outlook

### 6.1. Metagenomics and Microbiome-Informed Management

Shotgun metagenomic and full-length 16S rRNA sequencing permits characterisation of the entire microbial community rather than a single target organism [9,76,77]. Margot et al. demonstrate the approach’s value concretely, identifying pioneer genera—*Comamonas*, *Caulobacter*, *Schlegella*, *Blastomonas* and *Methyloversatilis*—in the biofilm formation process and tracking succession over eight weeks [17]. They note that identifying pioneer organisms helping *Legionella* establish could support strategies interfering with colonisation [17].

Abdel-Nour et al. identify the same direction from the mechanistic side, noting that the role of other bacterial species in *Legionella* biofilm production—and the mechanism by which certain species promote growth while others inhibit it—is an area of increasing interest [18]. Bai et al. report that metagenomic next-generation sequencing achieves sensitivity greater than PCR and culture and can simultaneously identify co-infecting pathogens within 48 h, though at high cost and with difficulty comparing read counts between samples and facilities [6].

Barriers include cost, bioinformatic capacity, difficulty distinguishing correlation from causation in observational building datasets, and the absence of standardised sampling and analysis protocols enabling cross-study comparison.

### 6.2. Whole-Genome Sequencing for Outbreak Attribution

WGS has substantially improved source attribution relative to sequence-based typing [78,79]. The principal constraints are the requirement for a cultured isolate—reintroducing culture’s recovery limitations at the critical investigative step—and the frequent absence of clinical isolates, since UAT-based diagnosis yields no organism. The magnitude of this problem is now quantifiable: with approximately 82% of European cases confirmed by UAT [6], the great majority of clinical cases generate no isolate for comparison with environmental strains. Increasing use of direct-from-specimen metagenomic sequencing may partially resolve this [6].

### 6.3. Quantitative Microbial Risk Assessment

QMRA links environmental concentration to expected infection burden through aerosolization, dispersion, inhalation, deposition and dose–response modelling [80,81,82]. Its promise is to place action thresholds on a defensible quantitative footing rather than historical convention. Substantial ambiguity remains, since dose–response parameters draw chiefly from guinea pig inhalation studies of limited relevance to heterogeneous human susceptibility; aerosolisation efficiency varies by orders of magnitude among fixture types; and the relationship between bulk water concentration, biofilm reservoir and aerosol concentration is not well constrained. Ortiz et al. reviewed mechanistic models of *Legionella* in building water systems and concluded that model predictions can be integrated with QMRA to inform decision-making while identifying hydraulics, materials, nutrients, temperature and disinfection as the factors most in need of better parameterisation [22].

Two findings in this review add parameters that current QMRA models do not carry. First, amoeba-associated and vesicle-packaged *Legionella* may have infectivity substantially different from that of planktonic cells [5,18]. Second, and more tractably, the VBNC fraction has a measurable infectivity ratio: Dietersdorfer et al. estimate 700–6300 infective cells per 10^7^ starved VBNC cells against 40,000–65,000 per 5 × 10^6^ culturable cells [5]. A QMRA that treats a culture-negative system as zero-risk is making an assumption these data contradict.

### 6.4. Sensor Networks, Digital Twins, and Machine Learning

Instrumenting building water systems with networked temperature, flow, pressure and residual sensors generates data at temporal resolution unavailable to any sampling regime [19,83]. Coupled with hydraulic and thermal models, this permits the identification of stagnation zones that as-built drawings and intuition miss, simulation of intervention scenarios, and anomaly detection.

Realistic assessment must acknowledge the limitations: models trained on culture-based outcomes inherit culture’s VBNC blindness; datasets are small, site-specific and rarely externally validated. Xu et al. illustrate both the promise and the constraint: their machine learning model predicts *Legionella* occurrence in premise plumbing from routinely measured, low-cost physicochemical variables, which would strengthen the Tier 1 layer described in Section 3.6 without additional microbiological sampling, but it is trained against conventional detection endpoints and therefore inherits their limitations [84]. The near-term value is more plausible in operational optimisation—ensuring temperature and residual targets are actually met throughout the system, as Marchesi et al.’s dosing-verification result implies [47]—than in replacing microbiological measurement.

### 6.5. Novel Control Concepts

Several approaches remain at the proof-of-concept stage. Abdel-Nour et al. highlight nanoparticles as able to disrupt *Legionella*–amoeba interactions and biofilm structure, as well as to clear the organism from mixed-species biofilms [18]—though environmental release and toxicity questions are unsettled. Bacteriophage and phage-derived enzymes offer high specificity, sparing the protective background community, with delivery into biofilm and regulatory approval as the major obstacles. Anti-biofilm and anti-quorum-sensing agents disrupt matrix formation or intercellular signalling rather than killing directly, potentially reducing selective pressure for tolerance; Abdel-Nour et al. note that which quorum sensing systems regulate *Legionella* biofilm formation remains unknown [18]. Amoeba-targeted control addresses the replication niche directly and is conceptually compelling, given the ecology described in Section 2, but remains essentially undeveloped as a practical intervention.

### 6.6. Priority Research Gaps

1. Measure the VBNC-to-culturable ratio in operating building water systems. Alleron established that the ratio can approach totality after disinfection in a laboratory biofilm [31]; Dietersdorfer established that VBNC cells retain infectivity at roughly 1% efficiency [5]. Neither tells us the ratio in a functioning hospital. With respect to the analysis presented here, this is among the most consequential unmeasured quantities in the field, and it is measurable with existing methods.
2. Evaluate viability-corrected ddPCR for *Legionella* in premise plumbing. ddPCR removes the calibration-curve circularity [44] and resists false positives from degraded DNA [38] but cannot discriminate viability [39]. Coupling it with PMA pretreatment is a logical next step and appears not to have been systematically assessed in building water systems.
3. Monitor a shock-disinfection cycle with three parallel measurements—culture, ddPCR, and viability-corrected molecular measurements—to separate culturable, VBNC-intact, and dead-but-detectable populations, thereby distinguishing incomplete biofilm penetration, protozoal protection [18], and VBNC conversion as mechanisms of rebound.
4. Establish molecular action levels empirically. Multi-site investigations linking qPCR and ddPCR measurements with culture-based detection and human disease incidence are needed to validate and define appropriate GU/L thresholds.The metrological groundwork exists [44]; the epidemiological work does not and is complicated by UAT-driven ascertainment bias [6].
5. Report serogroup distribution as standard in disinfection studies. Marchesi et al.’s serogroup 1 enrichment under chlorine dioxide [47] would have been invisible in a study reporting only total counts.
6. Conduct independent, multi-site, concurrent-control disinfectant evaluation. Liu et al. demonstrate that concurrent control is achievable within a single institution [55]; nothing prevents the extension of that design across institutions.
7. Characterise non-*pneumophila* species and non-serogroup-1 burden. Margot et al. found *L. rubrilucens* and *L. geestiana* with distinct colonisation dynamics [17]; *L. micdadei* behaved differently from *L. pneumophila* under starvation [5]. With UAT detecting only serotype 1 and confirming 82% of European cases [6], the true burden is unknown rather than small.
8. Resolve the biofilm-versus-protozoa confound. Abdel-Nour et al. state that whether disinfection resistance derives from biofilm structure, amoebal association, or both cannot currently be determined [18]. This is answerable experimentally and would materially change the control strategy.
9. Broaden dPCR platform evaluation. Both *Legionella* ddPCR studies appraised here used Bio-Rad systems [38,44], consistent with the wider water-microbiology literature [39]. Cross-platform reproducibility is unestablished, and one key assay remains proprietary [44].

## 7. Conclusions

This review has brought together three bodies of evidence that are ordinarily considered separately—the mechanistic ecology of *Legionella* in premise-plumbing biofilms, the analytical performance of culture-based and molecular detection, and the design quality of the comparative-effectiveness literature on secondary disinfection—and has examined what follows when they are read against one another.

Four findings emerge that are reasonably well supported by the primary literature appraised here. First, *Legionella* can colonise new plumbing biofilm within weeks rather than only after biofilm maturation, so newly commissioned and recently renovated systems warrant specific attention [17]. Second, monochloramine at concentrations used in practice can abolish culturability while leaving total genomic units and a substantial membrane-intact fraction largely unchanged, and starved VBNC cells retain infectivity for primary human macrophages at approximately 1% of the efficiency of culturable cells [5,31]. Third, shock disinfection—thermal or chemical—produces, at best, transient reductions, a conclusion on which independent groups and a recent meta-analysis agree [1,2,21,47]. Fourth, sustained controls with verified delivery at the point of use, whether chemical, thermal or point-of-use physical barriers, outperform intermittent intervention at costs that differ by more than an order of magnitude between modalities [20,21,47].

Three gaps are correspondingly well defined. The ratio of VBNC to culturable cells in operating buildings has not been measured, although existing methods would permit it. Viability discrimination has not been coupled to droplet digital quantification and validated in building water, and no jurisdiction has established action levels in the units that the most informative methods report because the epidemiological work linking genomic units to disease incidence has not been done. These gaps, rather than any deficiency in detection capability, define the priorities set out in Section 6.6.

The remaining argument developed in this review is interpretive rather than evidential and is presented as such.

The organism itself is comparatively well characterised. It is an amoeba-adapted intracellular parasite [5,18,27] that can colonise new plumbing biofilm earlier than has commonly been assumed [17], persists through disinfection in a state that the reference verification method does not detect [18,31], and retains—at reduced but non-zero efficiency—the capacity to infect human macrophages after a year of starvation under laboratory conditions [5].

**Figure 2:**
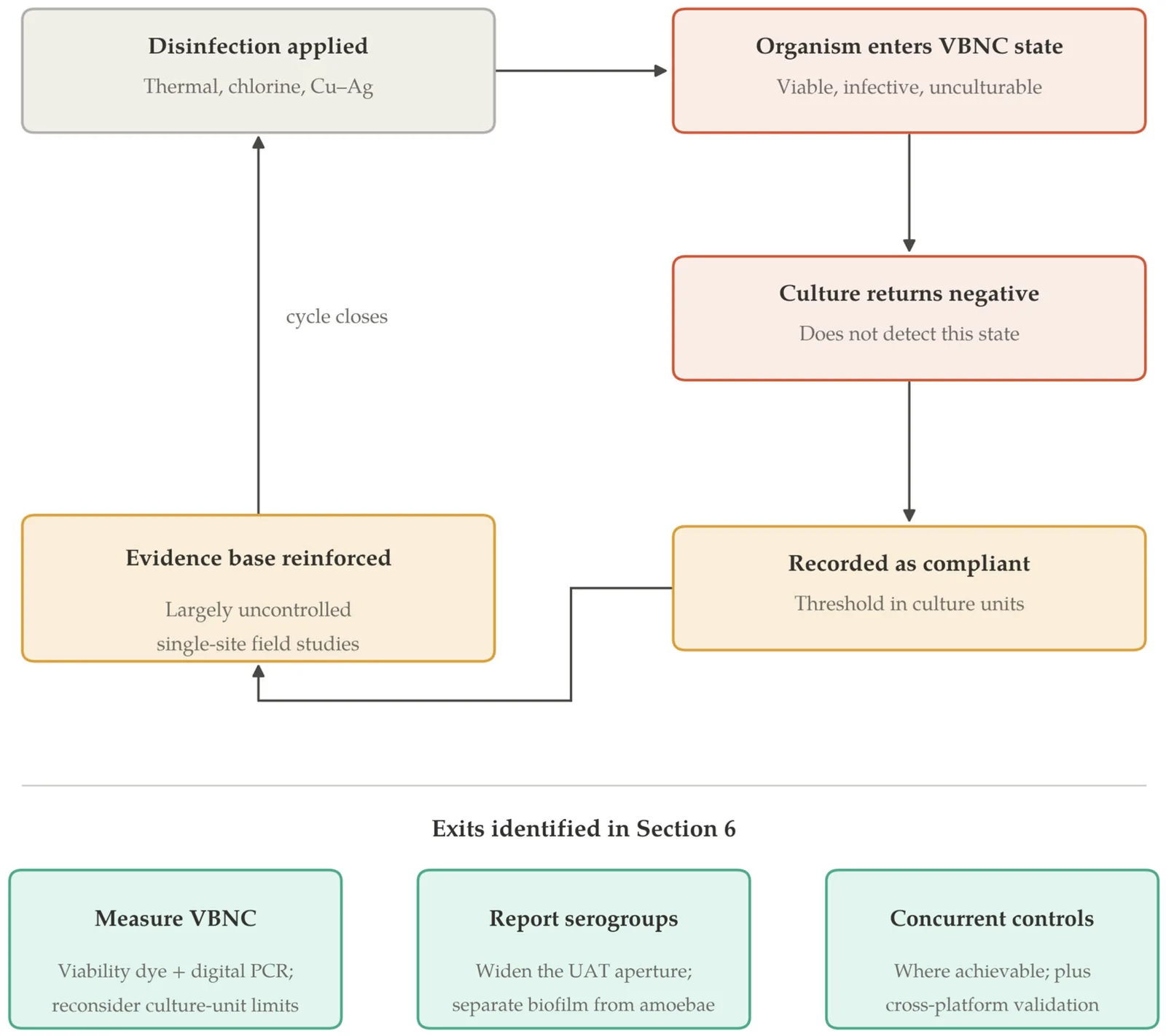
The closed verification loop in *Legionella* control practice. Box colors indicate stage: grey marks the initiating action (disinfection); red marks the detection failure (VBNC state entered and missed by culture); amber marks the resulting false-compliance loop (recorded as compliant, reinforcing the evidence base); green marks the exits proposed in Section 6.

Currently, key components of the control literature rely on single-site observational studies, depend on verification assays with specific sensitivity boundaries regarding non-culturable populations, and evaluate performance using regulatory units tied exclusively to traditional culture methods [1,2]. Recent systematic reviews and meta-analyses have begun to address the first of these limitations [20,21]. Figure 2 summarises this structure as interpreted here.

Detection technology has meanwhile advanced considerably. ddPCR quantifies without a calibration curve [44], resists inhibitors and degraded DNA [38,39], and achieves limits of detection in the low single figures of genomic units per reaction [44]. But it cannot distinguish viable from nonviable organisms [39], and no jurisdiction sets action levels in the units it reports. The capability gap has narrowed considerably; the interpretive gap appears to have narrowed less.

The surveillance data on which the entire risk picture rests carry a parallel problem. With approximately 82% of European cases confirmed by a serogroup-1-specific urinary antigen test of 55–80% sensitivity [6], the epidemiology the field is trying to control is observed through a substantially narrowed aperture.

These observations do not identify failures of individual studies. Stout and Yu correctly note that randomised trials of hospital water disinfection are impractical [2]. Marchesi et al. conducted a ten-year field programme and reported both their null result and their serogroup finding [47]. Alleron et al. identified the limits of their own resuscitation data [31]. Dietersdorfer et al. stated that the human-disease implications remain undetermined [5]. Abdel-Nour et al. stated that the biofilm-versus-protozoa confound could not be resolved with the available evidence [18]. Falzone et al., Tiwari et al. and Baume et al. each stated the in vitro, viability or proprietary-assay limitations of the methods they described [38,39,44]. The individual studies are, for the most part, appropriately qualified. The argument advanced here concerns the aggregate rather than any component: the high operational confidence surrounding certain *Legionella* control strategies would benefit from broader validation through multi-site, comparative field studies. Readers who weight the consistency of field outcomes more heavily than design quality may reasonably reach a less critical assessment, and the evidence reviewed here does not exclude that reading.

Disinfection may drive a fraction of the population into the viable-but-nonculturable state (Section 2.4); culture, the reference verification method, does not detect that state (Section 3.1); the resulting negative result is recorded as compliance against a threshold expressed in culture units (Section 5.6); and that compliance is absorbed into an evidence base composed largely of uncontrolled single-site before–after studies (Section 4.5), which, in turn, supports the original disinfection strategy. No step in the cycle is individually unsound, and most cited investigators state their own limitations explicitly. This schematic illustrates potential self-referential dynamics within routine verification frameworks when traditional culture remains the exclusive monitoring endpoint. This schematic represents the author’s interpretation of the literature rather than an empirically derived model.

The research priorities that follow are specific rather than conceptual: measure the VBNC fraction in real systems, couple viability discrimination to the better quantification technology now available, report serogroups, disentangle biofilm from protozoal protection, use concurrent controls where they are achievable, and reconsider the expression of regulatory thresholds in units that the mechanistic literature indicates are least informative under precisely the conditions the thresholds exist to govern.

## Figures and Tables

**Table 1:** Detection technologies for *Legionella* in water systems, with evidence evaluation.

Method	Turnaround	Sensitivity	VBNC Detection	On-Site Feasibility	Evidence Quality
Culture (ISO 11731)	7–14 d	Environmental: moderate; acid pretreatment suppresses competing flora at cost to target recovery [37]. Clinical: 60–80% sens., ~100% spec. [6]	None, by definition [18,31,39]	Very low	Extensive; its principal deficiency, however, cannot be quantified by the method itself; only method identifying all species and serotypes [6]
Serological assays	Short	78–90% sens., ~99% spec. [6]	n/a (host response)	Low	Retrospective and confirmatory use; antibodies may be shared across serogroups [6]
UAT	15 min	55–80% sens., 100% spec. [6]	n/a (antigen)	High (clinical)	Detects serotype 1 only; confirms ~82% of European cases [6]—a major contributor to ascertainment bias
qPCR	3–6 h	17–100% clinical sens., 95–100% spec. [6]; C_t_ 31.10 at 10 CFU/mL [38]	Yes, without viability discrimination [31,39]	Low–moderate	Good; interpretation contested False positives from degraded DNA post treatment [38]. Widely fluctuating sensitivity [6]
Viability qPCR (PMA)	4–7 h	High	Yes, membrane-integrity-based	Low–moderate	Moderate; dye penetration into biofilm aggregates imperfect [42,43]
ddPCR	4–8 h	Highest; LOD 2.5 GU/reaction in duplicate, LOQ 15 GU/reaction [44]; r^2^ 0.84 vs. 0.52 for qPCR [38]	No—quantifies genomes irrespective of viability [39]	Low; high capital and consumable cost [39]	Strongest analytical evidence located, but both *Legionella* studies used Bio-Rad platforms [38,44]; one assay proprietary [44]; dynamic range only 3–4 log_10_ [39]
LAMP (isothermal)	≤2 h	5.2–52 copies/reaction; 100% sens. vs. culture in 107 environmental samples [37]; >90% spec. clinically [6]	Yes; no viability discrimination	High—no thermal cycler required [37]	Single-laboratory validation; spec. 91.6–93.3% in environmental matrices [37]; not promoted in clinical practice [6]
mNGS	≤48 h	Greater than PCR and culture [6]	Yes; no viability discrimination	Very low	Unbiased; identifies co-infections; high cost; read counts not comparable between facilities [6]
Flow cytometry	15–30 min	High for total/intact counts; not species-specific [17]	Yes (membrane integrity)	Moderate	Established as a community indicator, not a *Legionella* assay

Note: Clinical sensitivity and specificity figures from Bai et al. [6] refer to diagnosis of *Legionella* pneumonia, not environmental monitoring and are not directly transferable. “n/a” in the VBNC Detection column indicates the method does not directly assess viability (serological assays detect host antibody response; UAT detects antigen) and so cannot be classified for VBNC detection.

**Table 2:** Effectiveness and cost of *Legionella* control methods.

Method	Δ% Positive Points	Δ% Points > 10^4^ cfu/L	Total €/Year per 100 Water Points
Chlorine dioxide (continuous)	−46.2	−82.3	3063
Shock superheating (monthly)	+30.5	−17.9	3710
Shock hyperchlorination (monthly)	−3.8	−83.5	7526
Electric boiler	−94.3	−100	8000
Point-of-use filter	−100	−100	93,600

Note: Source: Marchesi et al. [47]; Xi et al. [20], Table I. Costs are in 2010 euros for a specific Italian hospital and are not transferable without adjustment.

**Table 3:** Secondary disinfection approaches, graded by the design quality of the best evidence located.

Approach	Best Evidence Located	Design Quality	What It Supports	What It Does Not
Copper–silver ionisation	Stout & Yu [2]; Liu et al. [55]	Survey, no controls [2]; concurrent-control field study [55]	Sustained reduction in culture positivity; biofilm ion accumulation gives residual effect [55]	Disease prevention (no controlled clinical outcome); efficacy against VBNC
Chlorine dioxide	Marchesi et al. [47]	Single-site, 3 yr, dose–response quantified, no concurrent control	Dose-response relationship; 0.3 ppm at outlet gives <100 cfu/L [47]; lowest cost [47]	Eradication; protection during dosing interruption [47]; freedom from serogroup selection [47]
Monochloramine	Alleron et al. [31]; Marchesi et al. [47,49]; Farina et al. [52]	Laboratory biofilm model [31]; preliminary field, *n* = 20 [47]; 10 yr single-site field programme, no concurrent control [52]	Induces total loss of culturability at ≥1 mg/L [31]; sustained field control over ten years alongside a water safety plan [52]	Killing—GU unchanged, ~29% membrane-intact at 15 d, ~10% esterase-active at 145 d [31]; does not eradicate from biofilm [18]
Thermal shock	Marchesi et al. [47]; Lin et al. [1]	Repeated single site; independent concurrence; meta-analytic support [21]	No durable reduction—return to baseline within one month [47]	Any sustained control; amoebal cysts survive heat [18]
Hyperchlorination	Marchesi et al. [47]; Lin et al. [1]	Repeated single site; independent concurrence; meta-analytic support [21]	Transient reduction only; rebound at 2 months [47]	Sustained control; cost-effectiveness [47]
Point-of-use filtration	Marchesi et al. [47]	Small *n* (16 samples); result unambiguous within that sample	100% negative at filtered outlets [47]	System decontamination; affordability at scale (~€93,600/100 points/yr) [47]
Electric boilers (POU heating)	Marchesi et al. [47]	Field, *n* = 57 samples	Near-total control at >58 °C; 5/57 positive, all at 40–42 °C [47]	Applicability beyond targeted high-risk rooms
UV	Lin et al. [1]; Abdel-Nour et al. [18]	Review-level evidence only; no primary study appraised here	Point-of-entry protection of a defined branch	System-wide control—no residual; less efficacious than chlorine derivatives; sediment biofilms shield from UV [18]

## Data Availability

No new data were created or analyzed in this study. Data sharing is not applicable to this article.
